# Precision cut lung slices: a novel versatile tool to examine host–pathogen interaction in the chicken lung

**DOI:** 10.1186/s13567-019-0733-0

**Published:** 2020-01-10

**Authors:** Karen Jane Bryson, Damien Garrido, Marco Esposito, Gerry McLachlan, Paul Digard, Catherine Schouler, Rodrigo Guabiraba, Sascha Trapp, Lonneke Vervelde

**Affiliations:** 1 Division of Infection and Immunity, The Roslin Institute, Royal (Dick) School of Veterinary Studies, University of Edinburgh, Easter Bush, Midlothian, Edinburgh, Scotland EH25 9RG UK; 2 Division of Developmental Biology, The Roslin Institute, Royal (Dick) School of Veterinary Studies, University of Edinburgh, Easter Bush, Midlothian, Edinburgh, Scotland EH25 9RG UK; 3 INRAE, Université de Tours, UMR ISP, Centre Val de Loire, 37380 Nouzilly, France

## Abstract

The avian respiratory tract is a common entry route for many pathogens and an important delivery route for vaccination in the poultry industry. Immune responses in the avian lung have mostly been studied in vivo due to the lack of robust, relevant in vitro and ex vivo models mimicking the microenvironment. Precision-cut lung slices (PCLS) have the major advantages of maintaining the 3-dimensional architecture of the lung and includes heterogeneous cell populations. PCLS have been obtained from a number of mammalian species and from chicken embryos. However, as the embryonic lung is physiologically undifferentiated and immunologically immature, it is less suitable to examine complex host–pathogen interactions including antimicrobial responses. Here we prepared PCLS from immunologically mature chicken lungs, tested different culture conditions, and found that serum supplementation has a detrimental effect on the quality of PCLS. Viable cells in PCLS remained present for ≥ 40 days, as determined by viability assays and sustained motility of fluorescent mononuclear phagocytic cells. The PCLS were responsive to lipopolysaccharide stimulation, which induced the release of nitric oxide, IL-1β, type I interferons and IL-10. Mononuclear phagocytes within the tissue maintained phagocytic activity, with live cell imaging capturing interactions with latex beads and an avian pathogenic *Escherichia coli* strain. Finally, the PCLS were also shown to be permissive to infection with low pathogenic avian influenza viruses. Taken together, immunologically mature chicken PCLS provide a suitable model to simulate live organ responsiveness and cell dynamics, which can be readily exploited to examine host–pathogen interactions and inflammatory responses.

## Introduction

Organotypic tissue slices have been prepared from a number of organs for decades, with the advent of automated slicers allowing reproducible thin sections to be generated from tissues [1]. Precision-cut lung slices (PCLS) have been obtained from a range of species including mice [2] guinea pigs [3], sheep [4], pigs [5, 6] and humans [7]. PCLS were originally used in toxicology studies; however, the advantages of the model have allowed it to be applied to wider applications. The major advantage of the model is that it maintains the 3-dimensional (3D) architecture of the lung, which is lost in in vitro cultures of cells isolated from the lung. This 3D tissue model faithfully reflects the natural and relevant microenvironment of the respiratory tract. The PCLS model has the additional advantage that tissue from a single animal can generate tens to hundreds of slices, having the dual benefit of reducing experimental error by generating a large number of replicates, and reducing the number of animals required to test a hypothesis, thus meeting the 3Rs principle. As PCLS can remain viable in culture for a number of weeks [2, 4], this allows dynamic time course studies to be carried out in the same tissue. PCLS thus provide a timely and ethically acceptable platform for large-scale screening of lung tissue physiological and pathological responses.

As viable immune cells, including macrophages, neutrophils, DCs and T cells, have been identified in mammalian PCLS [8], PCLS have been used for studies on host–pathogen interactions, including viral infection and inflammatory responses [7, 9–11], and viral/bacterial co-infection [5]. Furthermore, PCLS are applicable to live, dynamic imaging of immune interactions [8, 12]. The 3D structure of the PCLS is of particular importance in elucidating the dynamic behaviour of the immune response in situ. Applying bio-imaging tools to PCLS renders capturing the initial and very early immune events including host–pathogen interactions much more accessible than would otherwise be possible in vivo.

The avian respiratory system, in contrast to the mammalian system, utilises a collection of air sacs to direct the airflow in the lungs, rather than a diaphragm [13–15]. Avian lungs thus do not expand and contract like mammalian lungs and are instead rigid, fixed at the thoracic walls. The air flow in the avian respiratory tract is unidirectional and requires two cycles of respiration to move through the entire system. Gas exchange takes place in air capillaries present in the parabronchial tissue. The unique anatomy of the avian respiratory tract and predisposition of poultry to respiratory infections, with numerous pathogens including avian influenza virus (AIV; reviewed in [16], and avian pathogenic *E. coli* (APEC; reviewed in [17])) entering and infecting the respiratory tract, highlights the importance of this organ for research. Immune responses in the avian lung are also of paramount importance, as the poultry industry routinely delivers sprayed or aerosolised vaccines to efficiently and cost effectively immunise large numbers of birds.

PCLS have previously been prepared from chicken embryos to assess the tropism of Infectious Bronchitis Virus (IBV) for bronchial and parabronchial epithelial cells [18]. However, embryonic chicken lungs are physiologically undifferentiated and lack a developed immune repertoire. In the present study, we have generated and validated the use of PCLS from immunologically mature (≥ 4 weeks old) conventional and specific-pathogen free (SPF) chickens, including *CSF1R*-reporter transgenic chickens [19] in terms of optimal growth media, viability, microanatomy, inflammatory responses and susceptibility to infection. *CSF1R*-reporter transgenic chickens express either mApple or eGFP under the control of the *CSF1R* promoter and enhancer, therefore fluorescent protein is expressed in cells of the mononuclear phagocyte lineage (MNP), with high levels of expression in cells of the macrophage lineage, and low levels of expression in granulocytes [19–21]. PCLS prepared from the *CSF1R*-reporter transgenic chickens allowed the dynamic, real-time imaging of fluorescent MNP cells and their interactions with the lung stroma and fluorescently labelled pathogens including a derivative of APEC O1 serotype expressing eGFP. Imaging initial host–pathogen interactions in chicken PCLS therefore provides a powerful tool to visualize and elucidate these early, decisive infection events.

## Materials and methods

### Birds

Commercial Hy-Line Brown and *CSF1R*-eGFP or *CSF1R*-mApple transgenic birds [19] were hatched and reared in floor pens at the National Avian Research Facility, The Roslin Institute, Edinburgh (UK). Animals were reared under conventional conditions but were not vaccinated. The chickens were housed in groups and received food and water ad libitum. Animals were housed in premises licensed under a UK Home Office Establishment License (PEL 60/4604) in full compliance with the requirements of the Animals (Scientific Procedures) Act 1986. Breeding of transgenic chickens was carried out under the authority of Project License PPL 70/8940 and application of substances was conducted under PPL 70/7860 with the consent of The Roslin Institute Animal Welfare and Ethical Review Board. White Leghorn chickens from the PA12 outbred line were raised in closed breeding since 1968 (Aycardi and Schellenberg, 1970). PA12 birds were hatched and reared under SPF conditions at INRAE (Plate-Forme d’Infectiologie Expérimentale, PFIE, Nouzilly, France) in full compliance with the requirements of the French regional ethics committee number 19 (Comité d’Ethique en Expérimentation Animale Val de Loire). Food and water were provided ad libitum.

### PCLS

Eight-week-old Hy-Line Brown chickens and *CSF1R*-eGFP or *CSF1R*-mApple transgenic chickens, and 4-week-old SPF PA12 were culled by cervical dislocation and death confirmed by decapitation. The chicken carcasses were inverted, held in position with a clamp stand and the trachea located with minimal dissection of the area. The trachea was intubated, tied off to create a seal and the lungs inflated with molten, pre-warmed (41 °C) 30–40 mL of 2% ultra-low gelling point agarose (Sigma Aldrich, Irvine, UK). The whole carcass was chilled at 4 °C for 1–2 h to allow the agarose to set prior to isolating the lungs. The lungs were dissected and biopsies generated using an 8 mm disposable biopsy punch (Integra Life science services, Saint-Priest, France). The 8 mm biopsy of lung tissue was attached to the piston of a Compresstome™ VF-300 OZ (Precisionary, Natick, MA, USA) vibrating microtome with superglue and embedded in 6% ultra-low gelling point agarose, following which 500 µm slices were cut. Alternatively, the 8 mm biopsies of the inflated SPF PA12 lungs were cut into 200–300 µm slices using a Krumdieck MD6000 Tissue Slicer (Alabama Specialty Products, Inc., Munford, AL, USA). All PCLS were collected in chilled Hanks’ Balanced Salt solution (HBSS) (Life Technologies, Paisley, UK) before being transferred into a 24 well plate, washed 3 times in appropriate media and cultured in 500 µL. Three different media were tested to optimise culture conditions: (1) high glucose Dulbecco’s Modified Eagles Media (DMEM) supplemented with 100 U/mL penicillin 100 µg/mL streptomycin, 4 mM l-glutamine, 20 µg/mL gentamycin, and 1.25 µg/mL Amphotericin B (Thermo Fisher, Paisley, UK), (2) DMEM F-12 Nutrient mixture (Ham; DMEM/F12) supplemented with 100 U/mL penicillin, 100 µg/mL streptomycin, and 4 mM l-glutamine and (3) DMEM/F12 supplemented with 100 U/mL penicillin, 100 µg/mL streptomycin, 4 mM l-glutamine and 5% heat inactivated FCS (DMEM/F12/FCS). Media and supplements were all purchased from Thermo Fisher Scientific (Paisley, UK) unless otherwise stated. The PCLS were incubated at 41 °C and 5% CO_2_ for 1 h per wash. The PCLS were then cultured at 41 °C and 5% CO_2_ in the different media, hereafter referred to as DMEM, DMEM/F12 and DMEM/F12/FCS, replacing the media every 24 h, with the day of slice preparation defined as day 0 post slice.

### Viability assay

AlamarBlue cell viability assays (Thermo Fisher Scientific, Paisley, UK) were carried out as per manufacturer’s instructions. Briefly the supernatant was removed from the PCLS and replaced with 450 μL of either DMEM, DMEM/F12 or DMEM/F12/FCS and 50 μL of AlamarBlue added per well of the 24 well plate, before incubating for 1 h at 41 °C. The supernatant was then harvested and the fluorescence assessed by excitation at 530 nm and the emission read at 590 nm using a Synergy HT plate reader (Biotek, Winooski, VT, USA). The supernatant from the PCLS was replaced with fresh media and the PCLS maintained in culture at 41 °C and 5% CO_2_. The viability assessment was repeated up to 44 days of culture.

### Live/Dead cell imaging

Fresh, unfixed, PCLS from PA12 chickens were prepared as described above, kept in culture for 1, 2, 3, 6 or 7 days in DMEM or DMEM/F12/FCS and stained with the LIVE/DEAD^®^ Cell Imaging Kit (488/570; Thermo Fisher Scientific, Paisley, UK) as per manufacturer’s instructions. Briefly, PCLS cultured in 24 wells plates were washed and the LIVE/Dead reagent added to an equal volume of cell culture media for 2 h. Next, PCLS were directly imaged in the plates under an Axiovert 200 M inverted epifluorescence microscope (Zeiss, Munich, Germany) at 200× magnification. Images were captured with an Axiocam MRm camera (Zeiss). For the quantification of dead cells within the PCLS at each time-point, images were acquired using the AxioVision SE64 software (Zeiss). Next, red fluorescent cells (dead cells) were counted in representative images acquired from 3 to 4 randomly selected PCLS. The numbers of dead cells were assessed using the Fiji-ImageJ image processing software, which permits practicable cell counting [22].

### Luciferase-based cytokine reporter assays

Type I interferons (IFNs) and IL-1β production by PCLS were measured in the supernatants using luciferase-based Mx- or NFκB-reporter bioassays, respectively [23, 24]. The CEC32-Mx-Luc and the CEC32-NFκB-Luc reporter cell lines are quail fibroblast cell lines carrying the luciferase gene under the control of the chicken Mx promoter [24] or carrying an NFκB-activated luciferase reporter gene [23] respectively. CEC32-Mx-Luc and the CEC32-NFκB-Luc were kindly provided by Prof. P. Stäheli (University of Freiburg, Germany). CEC32 luciferase reporter cells were cultured in DMEM GlutaMAX™-I supplemented with 8% heat-inactivated FCS, 2% heat-inactivated chicken serum, 4.5 mg/mL d-glucose, 100 U/mL penicillin, 100 μg/mL streptomycin and 50 μg/mL geneticin (all purchased from Thermo Fisher Scientific, Paisley, UK) and grown in 25-cm^2^ flasks (Corning Life Sciences, Bedford, MA, USA) at 41 °C and 5% CO_2_. To perform reporter assays, CEC32-Mx or CEC32-NFκB cells were seeded at 2.5 × 10^5^ cells/well in 24 well plates and incubated overnight at 41 °C and 5% CO_2_. The next day, cells were incubated for 6 h with the diluted supernatants (1/5 of total volume in fresh media) from chicken PCLS supernatants. Medium was removed and cells were washed twice with Phosphate Buffered Saline (PBS). Cells were lysed using the cell culture lysis reagent according to the manufacturer’s instructions, and luciferase activity was measured using the Luciferase assay reagent and a GloMax-Multi Detection System (all purchased from Promega, Charbonnières-les-Bains, France). Data were expressed as IFN-I or IL-1β activity (fold increase as compared to control group, which received fresh cell culture media only).

### Griess assay

Production of nitrite, a primary breakdown product of nitric oxide (NO), was assessed in the supernatant of the PCLS culture using the Griess Reagent Kit (Promega, Southampton, UK) as per manufacturer’s instructions. A nitrite standard curve was prepared by serially diluting a concentrated nitrite solution in DMEM. Supernatant from the PCLS was harvested after 24 h of incubation over the duration of the culture. As a positive control, supernatant was harvested from PCLS incubated for 24 h with 1 µg/mL LPS (*E. coli* 055:B5 Sigma Aldrich) on day 8 post slice. The supernatant was stored at −20 °C until use in the assay. Absorbance was then read at 550 nm using a Multiskan Ascent plate reader (Thermo Fisher Scientific, Paisley, UK). The limit of detection was 2.5 μM (125 pmol) nitrite.

### IL-10 ELISA

Chicken IL-10 was detected by capture ELISA as described by Wu et al. [25] using anti-chicken IL-10 capture antibody, clone ROS-AV164, and biotinylated detection antibody, clone ROS-AV163. Plates were incubated with twofold serially diluted standards (recombinant chicken IL-10) or supernatant. The absorbance was read at 450 nm (650 nm as a reference). The standard was fitted by linear regression and final concentration measures determined using Graph Pad Prism 8. The limit of detection was 70 pg/mL IL-10.

### RNA extraction and cDNA synthesis

RNA was extracted by homogenising the fresh 8 mm biopsy of lung tissue or cultured PCLS using a pestle and mortar in a small volume of liquid nitrogen, then adding 350 µL of RLT lysis buffer (Qiagen, Manchester, UK) supplemented with β_2_-mercaptoethanol (Sigma, UK). The homogenate in lysis buffer was transferred to a Qiashredder column and RNA extracted using the RNEasy kit (Qiagen) as per manufacturer’s instructions. The RNA quality and quantity was assessed using a Nanodrop spectrophotometer ND-1000 (Thermo Fisher Scientific, Paisley, UK) and cDNA was synthesized from 156 ng of RNA using Superscript III reverse transcriptase (Life Technologies, Paisley, UK) with a random oligonucleotide primer as previously described [26]. cDNA was stored at −20 °C.

### Quantitative PCR

Quantitative PCR was performed using TaqMan Universal PCR Master Mix (Applied Biosystems, Thermo Fisher Scientific, Paisley, UK), EvaGreen dye (Biotium, Freemont, CA, USA) and the following custom oligonucleotide primers; iNOS forward 5′-CAGCGGAAGGAGACAAACAGAG, iNOS reverse 5′-AACTCTTCCAGGACCTCCAGG, IL-1β forward 5′-CAGCAGCCTCAGCGAAGAG, IL-1β reverse 5′-CTGTGGTGTGCTCAGAATCCA, 28S forward 5′-GGCGAAGCCAGAGGAAACT and 28S reverse 5′-GACGACCGATTTGCACGT C (Sigma Aldrich). Each reaction contained 2 μL of cDNA diluted 1:5 in RNase/DNase free water. Quantitative PCR was carried out using an Applied Biosystems 7500 Fast Real-Time PCR System with the following cycle profile: 2 min at 50 °C, 10 min at 95 °C, followed by 40 cycles with denaturing for 15 s at 95 °C, and annealing/elongation for 1 min at 60 °C. Melting curves were generated to confirm a single-PCR product for each reaction as previously described [26]. All reactions were performed in duplicate.

### Immunofluorescent staining

To determine the structural integrity and phenotype of the cells present, PCLS were examined by immunofluorescent staining. PCLS generated from the PA12 chickens were fixed for 4 h in a 4% paraformaldehyde (PFA) solution in PBS at day 1 post slice. Fixed PCLS were washed in PBS and then stored in PBS containing 0.01% sodium azide at 4 °C. Next, the slices were washed in PBS followed by 30 min incubation at room temperature in a permeabilisation solution (0.25% Triton X-100 in PBS). PCLS were then washed in PBS and incubated for 30 min at room temperature with a blocking solution (PBS with 10% Bovine Serum Albumin, BSA, Sigma-Aldrich, Irvine, UK). For specific staining, PCLS were incubated for 3 h at room temperature with rhodamine phalloidin (F-actin probe conjugated to the red–orange fluorescent dye, tetramethylrhodamine—TRITC, Abcam, Cambridge, UK), rabbit anti-von Willebrand Factor (vWF) polyclonal antibody (A0082, Dako, Santa Clara, CA, USA,), mouse anti-chicken actin monoclonal antibody (JLA20, DSHB, Iowa, IA, USA,), mouse anti-β-tubulin monoclonal antibody (MA5-16308, Thermo Fisher Scientific, Paisley, UK), mouse anti-chicken monocytes/macrophages monoclonal antibody specific for the mannose receptor MRC1L-B (KUL01, BioRad, California, CA, USA) or mouse anti-chicken CD45 monoclonal antibody (UM16-6, BioRad, California, CA, USA), in order to identify endothelial cells, cytoskeleton, phagocytes and leukocytes, respectively. Corresponding isotype controls were used as recommended by the manufacturer. All antibodies were titrated and used at the optimal dilution. The PCLS were washed in PBS, followed by a 2 h incubation with goat anti-mouse IgG (H + L) Alexa Fluor 488 or goat anti-rabbit IgG (H + L) Alexa Fluor Plus 594 (both from Thermo Fisher Scientific, Paisley, UK) at room temperature. Following further washes in PBS, the PCLS were incubated for 10 min at room temperature in the presence of Hoechst 33342 dye (Thermo Fisher Scientific, Paisley, UK), then washed in PBS and mounted on glass slides using a Lab Vision™ PermaFluor™ Aqueous Mounting Medium (Thermo Fisher Scientific, Paisley, UK) prior to imaging by confocal microscopy.

Influenza A virus infected PCLS were either stained with non-structural protein 1 (NS1)-specific rabbit antiserum [27] and goat anti-rabbit IgG (H + L) Alexa Fluor 488 (Invitrogen, Paisley, UK) or with mouse anti-Influenza A Virus nucleoprotein (NP) (AA5H, Abcam) and goat anti-mouse Ig-FITC (Southern Biotech, Birmingham, AL, USA). Pre-immune rabbit serum was used as NS1 staining control. The slices were washed in PBS with 1% FCS and then incubated with Hoechst 33342 dye. The slices stained for NP were washed in PBS and imaged in a 35 mm µ-dish (Ibidi, Grafelfing, Germany) by confocal microscopy. The slices stained for NS1 were prepared and imaged as described for the other antibodies.

### Imaging of PCLS

The PCLS isolated from *CSF1R*-eGFP or *CSF1R*-mApple transgenic birds were imaged floating in DMEM within a 35 mm µ-dish (Ibidi), weighed down with sterile 13 mm coverslips (Scientific Laboratory Supplies Ltd, Coatbridge, UK). Live images were captured using a Zeiss Live Cell Observer with the heated stage set to 37 °C and the imaging chamber set to 37 °C and 5% CO_2_. PCLS at 3 days post slice were incubated with either red fluorescent 1 μm latex beads (Thermo Fisher Scientific, Paisley, UK) or 5 × 10^9^ CFU/mL APEC O1-eGFP.

Images of PCLS were also captured using a Zeiss Axio Zoom.v16 in the wells of a 24 well plate at 7X or 16X magnification. Confocal images were captured using a Leica TCS P8 confocal microscope at 200X or 630X magnification and the LAS X software (Leica, Wetzler, Germany) or a Zeiss LSM710 inverted confocal microscope and Zen software (Zeiss).

### Avian pathogenic *Escherichia coli* growth

Avian pathogenic *E. coli* (APEC) strain O1 (serotype O1:K1:H7) expressing enhanced green fluorescent protein (APEC O1-eGFP) was obtained by transformation of APEC strain O1 with plasmid pFVP25.1 and cultured as described previously [28]. The APEC strain O1 strain was kindly provided by Prof. L. Nolan, Iowa State University, USA [29]. APEC O1-eGFP was grown for 20 h in Luria–Bertani broth supplemented with 100 μg/mL ampicillin (Sigma Aldrich) at 37 °C to reach stationary phase. Prior to use, eGFP expression by the bacteria was confirmed by streaking the bacteria on LB-agar plates, incubating overnight at 37 °C and examining the colonies under UV light.

### Influenza A virus infection of PCLS

Low pathogenic avian influenza (LPAI) viruses A/chicken/Italy/1067/1999 (H7N1; [30]) or A/Mallard/Marquenterre/Z237/83 (H1N1) at 10^6^ PFU were added to PCLS from Hy-Line-Brown (H7N1) or PA12 chickens (H1N1) for 1 or 3 h of incubation at 37 °C, respectively. The supernatant was then removed and replaced with DMEM for up to 48 h. The supernatant was harvested and stored at −80 °C and the slices fixed, stored, stained and imaged as described above.

### Viral titre

Serial tenfold dilutions of supernatant from virus-infected PCLS were incubated with confluent MDCK cells for 1 h in a standard plaque assay [31] using an 1.2% Avicel/DMEM overlay supplemented with 0.14% BSA (Sigma Aldrich) and 1 µg/mL TPCK treated trypsin (Worthington Biochemical Corporation, Lakewood, NJ, USA). Briefly, the diluted supernatants were aspirated and the cells incubated for a further 48 h in DMEM at 37 °C and 5% CO_2_. The cells were fixed in 4% formalin for 20 min at room temperature, stained with 0.1% toluidine blue or crystal violet at room temperature for 20 min, then washed in water. The cells were left to air dry and plaques enumerated.

### Statistical analysis

Where applicable, data are expressed as mean ± SD. Statistical analysis was performed by one way ANOVA with post hoc Tukey test, using Graph Pad Prism 8.0 software (GraphPad, San Diago, CA, USA). The number of slices examined is defined as “n”. Statistical analysis was only performed where 3 or more birds had been used to generate slices and significance was considered at *p* < 0.05.

## Results

### Viability of PCLS

**Figure 1:**
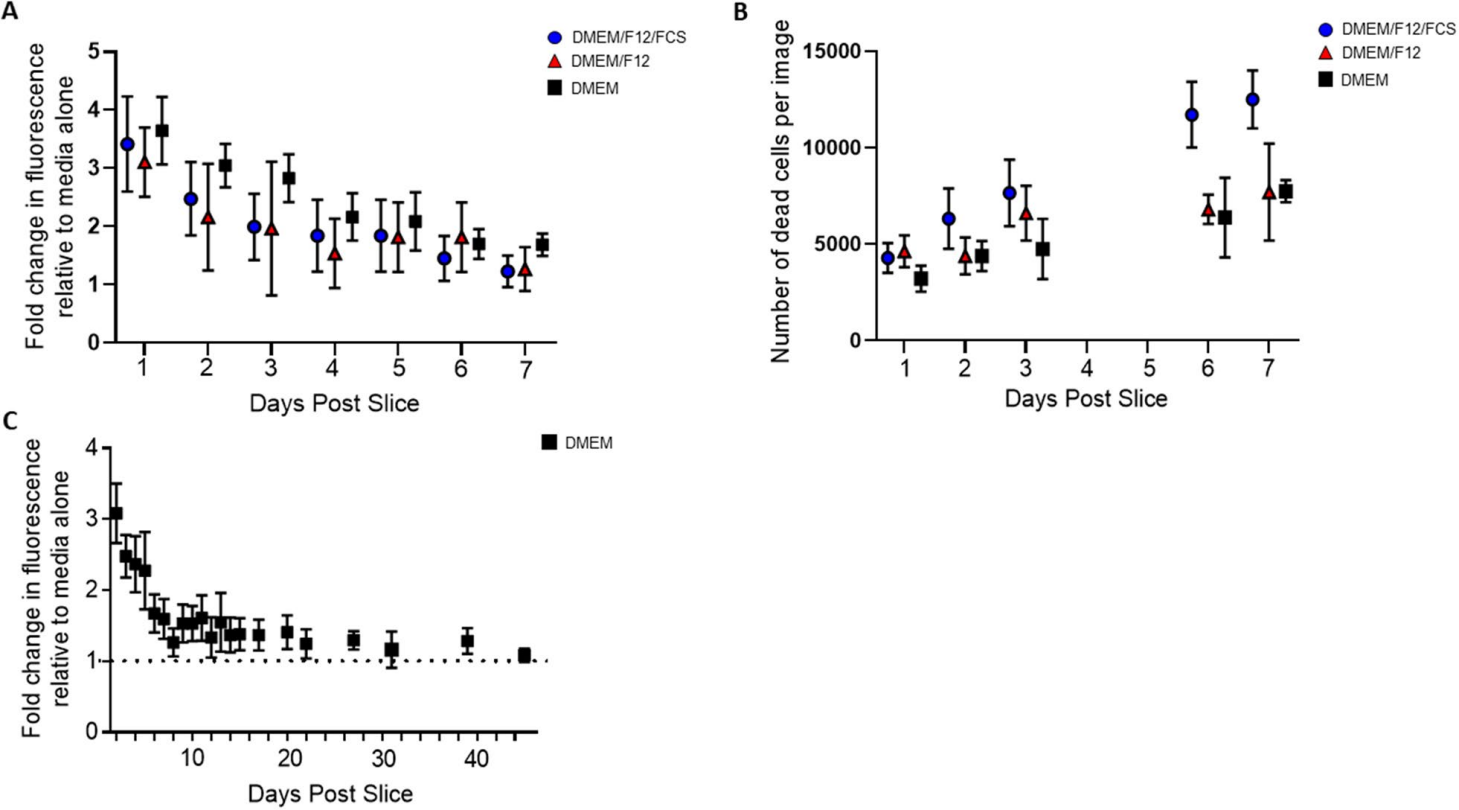
**PCLS viability over time.** PCLS viability was assessed by AlamarBlue assay (**A** and **C**) and Live/Dead staining (**B**). PCLS (500 μm) prepared from *CSF1R*-eGFP transgenic chickens were cultured for 7 days in either DMEM/F12/FCS (blue circle), DMEM/F12 (red triangle) or DMEM (black square) and viability was assessed using the AlamarBlue assay (**A**). *n* = 6–18 slices generated from 3 birds, data are represented as the mean ± SD. PCLS prepared from SPF PA12 chickens (200–300 μm) were cultured for 7 days in either DMEM/F12/FCS (blue circle), DMEM/F12 (red triangle) or DMEM (black square) and were stained with a Live/Dead kit and subsequently examined by epifluorescence microscopy (**B**). The number of dead cells were enumerated in images from 3 to 4 PCLS generated from 2 birds at different time-points using the Fiji-ImageJ image processing software. Data are expressed as the mean ± SD, per time-point of one representative experiment. *n* = 3–4 slices. The maintenance of cell viability in PCLS (500 μm) prepared from *CSF1R*-eGFP transgenic chickens was assessed in DMEM media by AlamarBlue assay (**C**). *n* = 16 slices generated from 2 birds, data are represented as the mean ± SD.

The viability of the PCLS was assessed using different culture conditions, to establish their nutrient and growth factor requirements over a period of 7 days. PCLS were cultured with DMEM, DMEM/F12 or DMEM/F12/FCS, with DMEM/F12 providing a broader range of inorganic salts, amino acids and vitamins than DMEM. PCLS cultured with DMEM, in the absence of FCS had a mean 30% greater cell viability, than those cultured in the nutrient and growth factor rich DMEM/F12/FCS at 3 days post slice (Figure 1A). These findings were confirmed in PCLS generated from the PA12 SPF chicken line, by Live/Dead staining (Figure 1B, Additional file 1). At 3 days post slice, PCLS cultured in DMEM/F12/FCS had a 24%, and 13.7% increase in dead cells compared with those cultured in DMEM and DMEM/F12 respectively. PCLS cultured in DMEM/F12/FCS had a mean 38% higher number of dead cells at 7 days post slice, compared with those cultured in DMEM. As a clear trend emerged, demonstrating that the presence of FCS had a detrimental effect on viability, and DMEM had an average of 12% less cell death than DMEM/F12 over the course of the culture period, serum free DMEM was established as the culture medium of choice.

The viability of PCLS in the established culture method was then assessed for > 40 days using an AlamarBlue assay (Figure 1C). The fluorescence intensity remained above the background detected in the absence of PCLS for 44 days in culture, suggesting that viable cells remained present in the slice for over a month in culture. The mean body temperature of chickens is 41 °C; however, as the lung is in constant contact with air at ambient temperature during inspiration, this is likely to reduce the temperature of the organ relative to core body temperature. Consequently, although data on the exact temperature of the avian lung is lacking, the effect of temperature on viability of the PCLS was considered. Incubation at 37 °C did not alter the viability relative to incubation at 41 °C over the course of the culture (Additional file 2). As a further means of assessing the long-term viability of the PCLS motile fluorescent mononuclear phagocytic cells (CSFR1^+^ cells) were identified by live cell imaging of PCLS prepared from *CSF1R*-eGFP transgenic chickens, at 21 days post slice (Additional file 3).

### Structural integrity and microanatomy of PCLS

**Figure 2:**
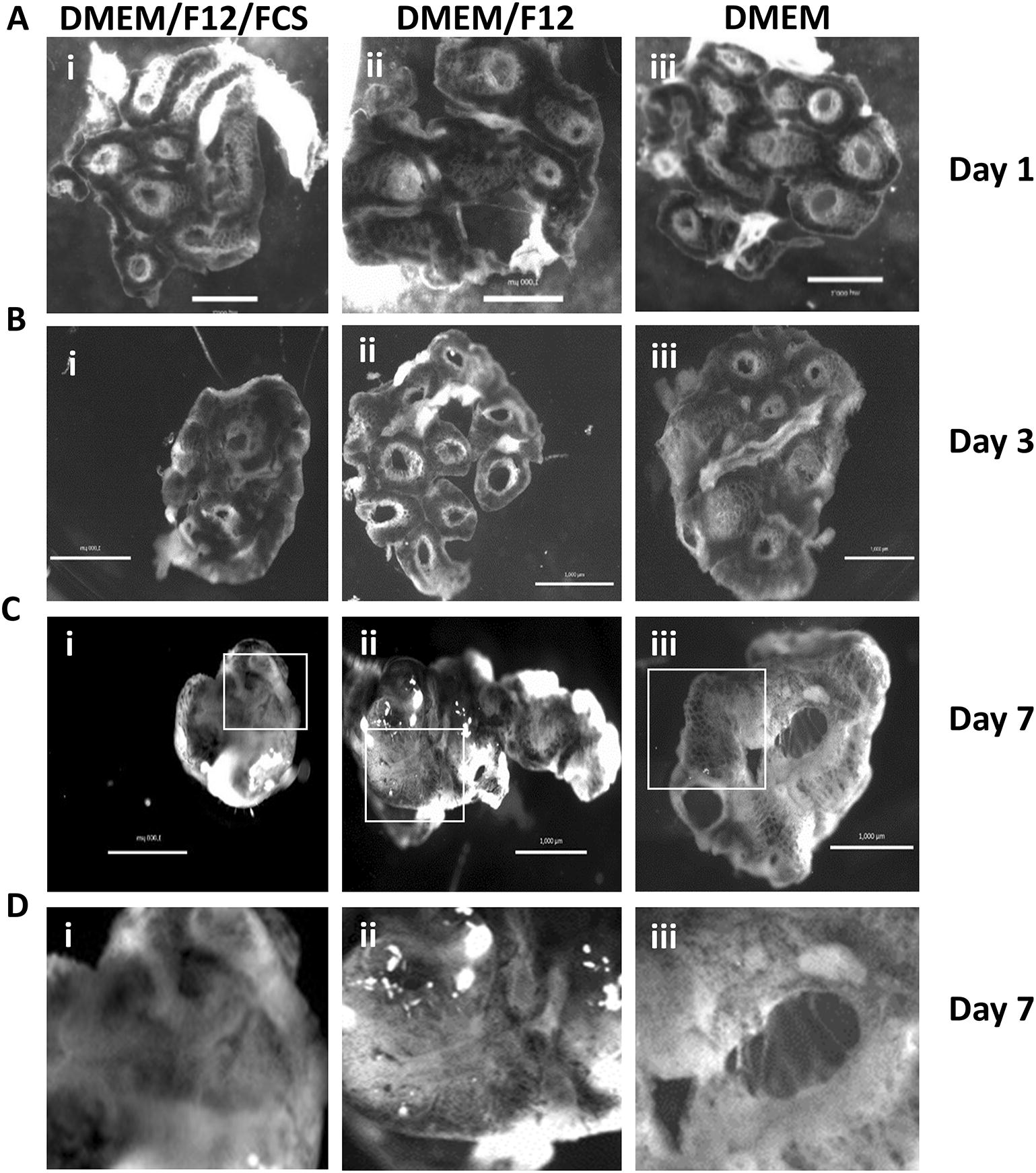
**Structural integrity of PCLS.** Bright field images were captured at 1 (**A**), 3 (**B**) and 7 (**C** and **D**) days post slice for PCLS (500 μm) prepared from *CSF1R*-eGFP transgenic chickens. The PCLS were cultured with DMEM/F12/FCS (i), DMEM/F12 (ii) or DMEM (iii). The white boxes (**C**, panels i, ii and iii) represent the area digitally enlarged (**D**, panels i, ii, iii respectively). Images were captured using the Zeiss Axio zoom V16. Images are representative of randomly selected PCLS from 3 individual birds. Scale bar = 1000 µm.

The structural integrity and microanatomy of the PCLS were investigated using whole mount imaging and immunostaining. In agreement with the viability assays, bright field imaging of *CSF1R*-eGFP transgenic chicken PCLS at 1, 3 and 7 days post slice (Figure 2), demonstrate that PCLS cultured in the absence of FCS were larger and maintained macrostructures such as air spaces to a greater extent than those cultured in the presence of FCS. The PCLS grown without FCS maintained a macroscopic structure resembling the intact lung at 7 days post slice. The presence of FCS resulted in noticeable shrinkage and curling of the tissue with apparent loss of lung parenchyma (Figure 2C, panel i and Figure 2D, panel i).

**Figure 3:**
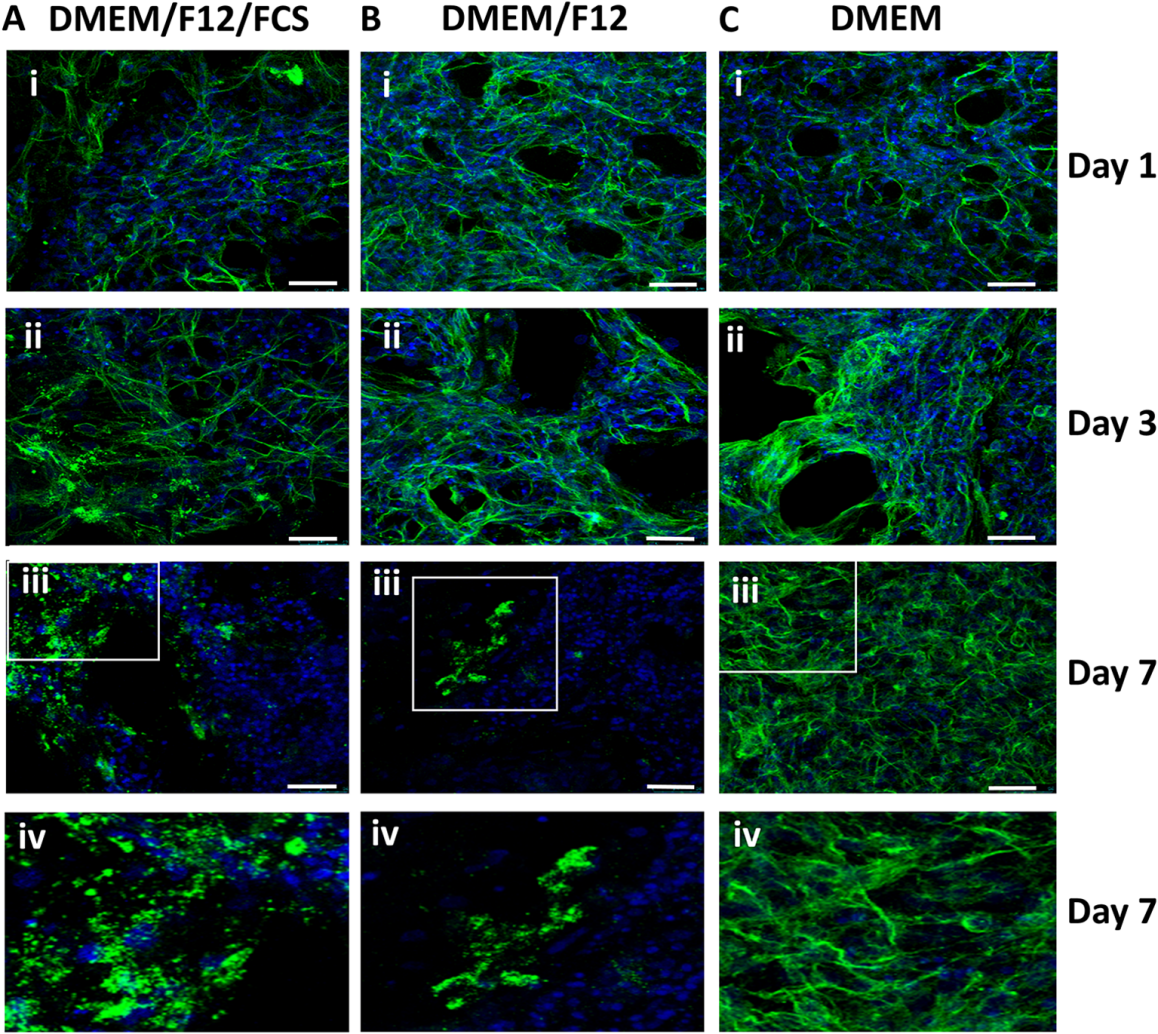
**PCLS cytoskeleton structure under different media conditions.** PCLS (200–300 μm) prepared from PA12 SPF chickens were cultured in DMEM/F12/FCS (**A**), DMEM/F12 (**B**), or DMEM (**C**) from 1 (i), 3 (ii) or 7 days (iii and iv) post slice. PCLS were fixed and β-tubulin filaments (green) and nuclei (blue) visualized. The white boxes (**A**, panel iii, **B**, panels iii and **C** iii) represent the area digitally enlarged (**A**, panel iv, **B**, panel iv, **C**, panel iv respectively). Images were captured using a Leica TCS P8 confocal microscope at ×630 magnification. Scale bars = 25 μm. Images are representative of 3 to 4 PCLS randomly selected from 2 birds per time point and condition.

In line with these observations, imaging of PCLS from SPF chickens immunostained with an anti-β-tubulin antibody to identify the cytoskeleton, revealed that as early as 1 day post slice and even more markedly at 7 days post slice, those PCLS cultured in the presence of FCS had marked areas of tubulin depolymerisation (Figure 3A). Depolymerisation was most notable in the borders of the slices and in the surrounding parabronchi, where several aggregates or loss of the tubulin network were evident. A similar pattern was observed for PCLS cultured in DMEM/F12 only at 7 days post slice (Figure 3B, panels iii and iv). However, these aggregates were not observed in PCLS cultured in DMEM, where the tubulin network was preserved up to 7 days post slice (Figure 3C, panels iii and iv), albeit with less organised structure relative to earlier time points. Isotype staining controls are provided in Additional file 4. As the viability and structural integrity of PCLS was relatively improved for those cultured in DMEM, henceforth DMEM alone was used in all further experiments.

**Figure 4:**
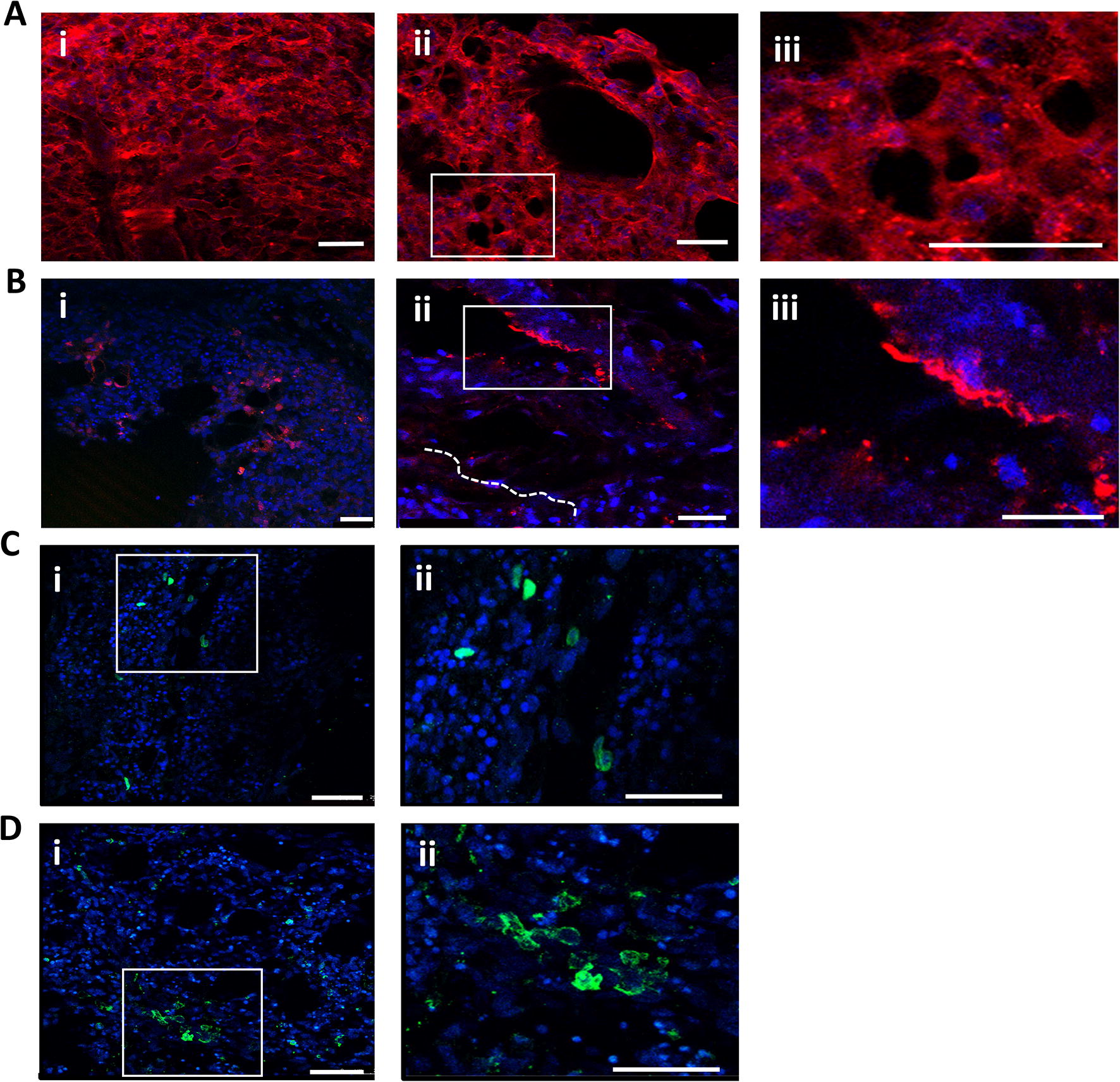
**Immunostaining of PCLS.** PCLS (200–300 μm) prepared from PA12 SPF chickens 1 day post slice were stained with a panel of antibodies and examined by confocal microscopy. Nuclei are represented in blue (DAPI). Actin cytoskeleton staining using rhodamine phalloidin (red) (**A**). Longitudinal section revealing boundaries of capillaries from the parabronchi (**A**, panel i); Transversal section revealing a parabronchus and air capillaries in greater details (**A**, panels ii and iii). The white box (**A**, panels ii) represents the area digitally enlarged (**A**, panels iii). von Willebrand Factor staining (red) (**B**), a marker of endothelial cells. Overall view of vWF-stained endothelial cells within the lung parenchyma (**B**, panel i); Longitudinal section (**B**, panel ii) with a schematic drawing of capillary networks beneath the lung parenchyma (dotted lines). The white box (**B**, panel ii) represents the area digitally enlarged (**B**, panel iii). Capillary ends are observed scattered in the tissue (**B**, panel ii); a capillary ramification close to a parabronchus and a capillary end in greater detail (**B**, panel iii). Longitudinal section showing MCRL1-B^+^ phagocytes (green) (**C**) within the lung parenchyma (**C**, panels i and ii), especially close to the septae. The white box (**C**, panel i) represents the area digitally enlarged (**C**, panel ii). Transversal section showing CD45^+^ leucocytes (green) within the lung parenchyma (**D**, panels i and ii), scattered in the atria and septae. The white box (**D**, panel i) represent the area digitally enlarged (**D**, panel ii). Images were captured using a Leica TCS P8 confocal microscope. Scale bars = 75 μm (**B**, panel i) and 25 μm. Images are representative of 3 to 4 PCLS randomly selected from 2 birds per staining strategy.

The PCLS microanatomy was visualised, with focus on the lung parenchyma, endothelial cells and the presence of resident leukocytes, using a selected panel of antibodies. For these experiments, PCLS were prepared from 4-week-old SPF PA12 chickens and actin filaments visualised to observe the overall chicken lung structure (Figure 4A). Distribution of actin filaments within a cell is an important determinant of cellular shape and structure. Actin staining with rhodamine phalloidin identified boundaries of capillaries in the parabronchi. This staining also provided a clear demarcation of parabronchial structures containing air capillaries, which are implicated in gas exchange and functionally comparable to mammalian alveoli (Figure 4A, panels ii and iii). Immunostaining with von Willebrand Factor (vWF), a canonical marker of endothelial cells, allowed the identification of blood capillaries (Figure 4B), together with small clusters of vWF-positive cells indicating a longitudinal cut of the capillary lumen (Figure 4B, panels ii and iii). The parabronchi form the gas exchange tissue of the avian lung, where air capillaries are laced with a network of blood capillaries. MRC1L-B^+^ macrophages (Figure 4C), and CD45^+^ leukocytes (Figure 4D) were visualised within the lung parenchyma, confirming the findings with PCLS obtained from *CSF1R*-eGFP transgenic chickens. MCR1L-B^+^ cells were identified close to the septae and an abundance of CD45^+^ leukocytes were observed scattered in the atria and septae (Figure 4C). Staining controls are provided in Additional File 5. In conclusion, our data show that the 3D architecture and cell heterogeneity of the lung slices are maintained in culture.

### PCLS responses to inflammatory stimulus

**Figure 5:**
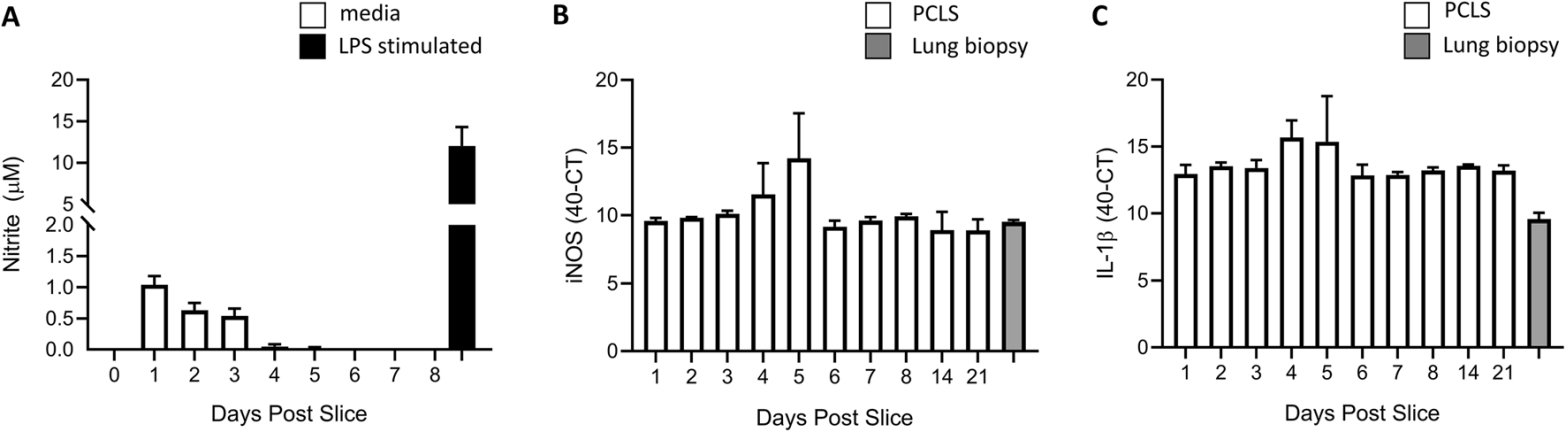
**Basal levels of inflammatory mediators in cultured PCLS.** Nitrite produced by PCLS (500 μm) prepared from *CSF1R*-eGFP transgenic chickens was assessed in the supernatant over 8 days of culture by Griess assay (white) (**A**). The supernatant was harvested every 24 h. As positive control, on day 7 a number of PCLS were stimulated with LPS for 24 h (black) and the supernatant harvested on day 8. *n* = 6–10 slices generated from 4 individual birds. iNOS (**B**) and IL-1β (**C**) mRNA levels were assessed by qPCR in the PCLS over 21 days post slice and normalised to 28S expression (white). *n* = 6 slices generated from 4 individual birds. iNOS and IL-1β levels were also assessed in a fresh lung biopsy sample (grey) that had not been sliced into sections or cultured with DMEM. *n* = 3–4 slices generated from 3 individual birds. All data are represented as the mean ± SD. *p < 0.05 all time points compared.

To examine any background inflammatory response of the PCLS induced by the slicing procedure, nitrite production was measured in the supernatant. Nitrite levels were below the limits of detection of the assay throughout the culture period, in the absence of stimulation (Figure 5A). The low levels of nitrite produced by the PCLS were consistent with the low levels of *iNOS* transcripts as assessed by qPCR, with no significant differences in the iNOS transcriptional expression levels (Figure 5B) detected in the PCLS over the duration of the culture. Similarly, no significant differences in IL-1β expression levels at any particular day post culture (Figure 5C) were detected by qPCR over the duration of the experiment. Furthermore, the PCLS levels of *iNOS* transcript were similar to those seen from a fresh biopsy of lung, which had not been processed or sliced into 500 µm sections as control for the effects of the processing and culture. The qPCR data thus confirmed that the background nitric oxide, and IL-1β levels in the absence of stimuli, were low, suggesting that inflammatory responses of the PCLS post slice were low, with no apparent alterations in this response post tissue slicing.

**Figure 6:**
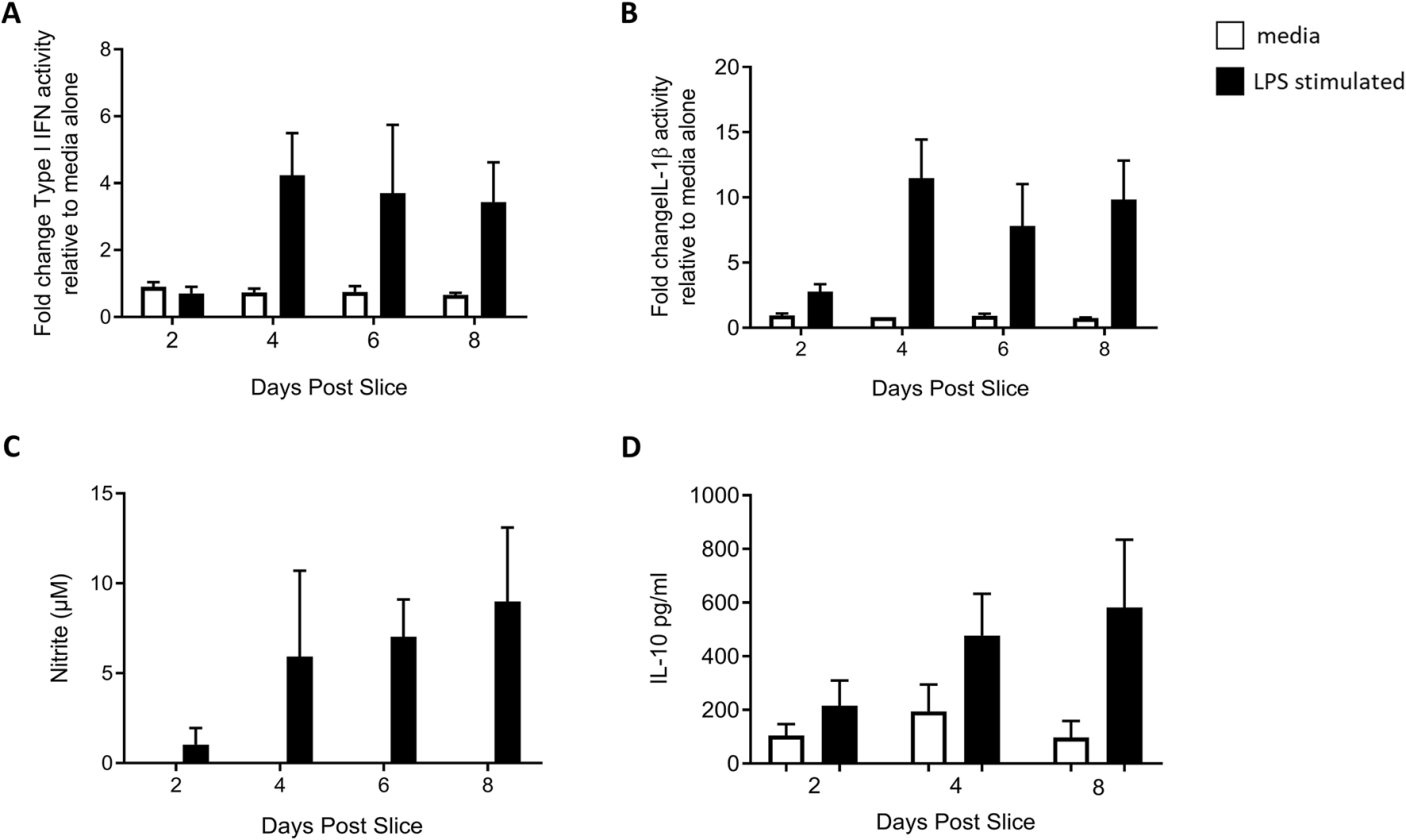
**PCLS responses to LPS.** PCLS (500 μm) prepared from from *CSF1R*-eGFP transgenic chickens and cultured in the presence (black) or absence (white) of LPS for 24 h on either day 1, 3, 5 or 7 post slice and supernatants harvested on day 2, 4, 6 and 8 post slice. IL-1β (**A**) and type I IFNs (**B**) production were measured in the supernatants using luciferase-based Mx- or NFκB-reporter bioassays. Results are expressed as fold change in luciferase activity relative to unstimulated PCLS. *n* = 3 slices generated from 2 individual birds. Nitrite produced by PCLS over 8 days of culture in response to LPS for 24 h was assessed in the supernatant by Griess assay (**C**). *n* = 6 slices generated from 4 individual birds. The concentration of IL-10 was assessed in PCLS supernatant by ELISA on day 2, 4 and 8 post slice (**D**). *n* = 6 slices generated from 4 individual birds. Data are represented as the mean ± SD.

The capacity to mount an inflammatory response was determined by measurement of IL-1β (Figure 6A) and type I IFNs (Figure 6B) bioactivity and nitrite production (Figure 6C) in the supernatants of PCLS following LPS challenge for 24 h at 1, 3, 5 or 7 days post slice. Accordingly, the supernatants were harvested on 2, 4, 6 and 8 days post slice. Substantial IL-1β and type I IFN responses to LPS stimulation were noted at 4 days post slice onwards, with over a tenfold increase in both IL-1β and type I IFN bioactivity in the supernatants of stimulated PCLS compared with unstimulated PCLS from 4 days post slice onward. Nitrite levels were also substantially increased in response to LPS stimulation, compared with PCLS cultured with media alone, from 6 days post slice, indicating that the PCLS were able to produce nitric oxide in response to an inflammatory stimulus. In order to assess the anti-inflammatory responses of the PCLS, IL-10 as a potential mediator of wound healing was measured in the supernatant by ELISA (Figure 6D). An increased IL-10 response to LPS was detected from day 4 that peaked at day 8 post slice. IL-10 production therefore conformed to the delayed LPS induced responses seen for IL-1β, type IFN and nitrite. Altogether the data confirmed that the chicken PCLS were capable of mounting a readily detectable inflammatory response to LPS stimulation.

### Host–pathogen interactions

In addition to assessing the inflammatory response, the phagocytic activity of mononuclear phagocytes in the PCLS was examined by live cell imaging at day 3 post slice. *CSF1R*-eGFP^+^ cells were imaged migrating (Additional file 6) and in the act of engulfing 1 μm latex beads (Additional file 7). Moreover, *CSF1R*-mApple^+^ cell dynamic interactions with APEC O1-eGFP were captured in the parabronchial tissue (Additional file 8). Thus the capacity of lung resident CSF1R^+^ cells to sample the environment and engulf foreign material is retained within the chicken PCLS maintained in culture.

**Figure 7:**
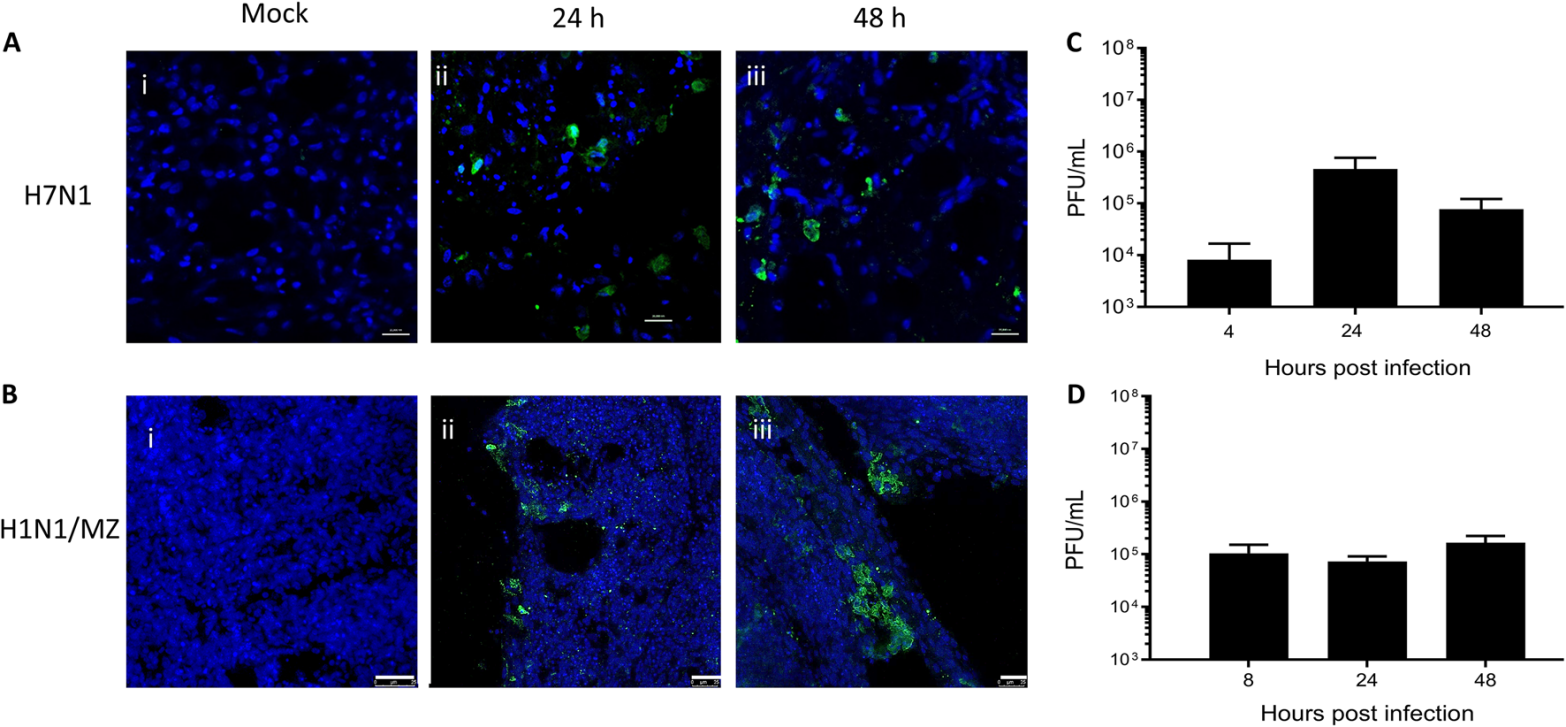
**PCLS are susceptible to low pathogenic avian influenza virus strains.** PCLS (500 μm) were generated from Hy-Line conventional (**A**) and PCLS (200–300 μm) were generated from PA12 SPF (**B**) chickens, infected with H7N1 (**A**) and H1N1 (**B**) LPAI virus strains, and stained for the viral proteins NP (**A**) and NS1 (**B**). Representative images showing uninfected PCLS (i) and virus-infected cells (green) within the PCLS at 24 (ii) and 48 (iii) hours post-infection. The nuclei are represented in blue. Images were captured by confocal microscopy at ×400 magnification. Scale bar = 10 µm (**A**) and 25 µm (**B**). *n* = 8 slices generated from 2 individual birds. Viral titre of supernatants harvested from H7N1-infected PCLS at 4, 24 and 48 h post-infection (**C**) and H1N1-infected PCLS at 8, 24 and 48 h post-infection (**D**). *n* = 8 slices generated from 2 individual birds, data are represented as the mean ± SD.

The permissiveness of the PCLS to viral infection was tested by infecting the slices with H7N1 (Figure 7A) or H1N1 LPAI virus strains (Figure 7B) followed by immunofluorescence detection of the viral NP and NS1 protein, respectively. The infection pattern detected by immunofluorescence staining was characterised by localised patches of viral protein (NP and NS1) positive foci rather than diffuse signals scattered in the parenchyma (Figure 7B). The H7N1 viral titre detected in the supernatant of the infected PCLS had a 100-fold increase at 24 h post-infection compared with 4 h post-infection, indicating that the influenza A virus was not only able to infect but also to productively replicate in the PCLS (Figure 7C). No change in titre was detected for the H1N1 virus (Figure 7D), which although able to infect PCLS, presented steady viral titres up to 48 h post-infection, a phenomenon that is reminiscent to findings obtained with this virus in other in vitro systems [32]. In conclusion, these results demonstrated the application of the chicken PCLS model to study bacterial and viral pathogens causing economically relevant respiratory diseases in poultry.

## Discussion

Research in the field of poultry sciences is continuously evolving with the development of some complex in vitro models to study chicken physiology, infection and inflammation, such as tracheal organ cultures [33], magnum organ cultures [34] and intestinal organoid cultures [35]. As for the avian lung, in vitro, monolayer cultures fail to provide tissue architecture and the full spectrum of cells present in vivo, therefore in vivo models have been prioritised in the past to study physiological functions and/or pathogenic challenges of the lung. PCLS provide an alternative that preserves both tissue architecture and the heterogeneous cell populations in the avian lung, including epithelial cells, endothelial cells, fibroblasts, mononuclear phagocytes and other leukocytes. The generation of the PCLS using two different tissue slicing apparatus from immunologically mature chickens (ranging from 4 to 8 weeks of age) from different chicken lines, indicates the reproducibility of the model and the ease of application to different laboratory settings, which allows for modifications to tailor it to specific scientific questions. The PCLS system thus provides a robust 3D functional model of the avian lung with extensive research potential.

After examining a range of culture conditions, FCS was found to be detrimental to the viability and structural integrity of the PCLS; hence, the PCLS from immunologically mature chicken were subsequently cultured in serum free conditions. The presence of viable cells in the PCLS was detected for over 40 days in serum free culture medium. This is consistent with the reports of ovine PCLS remaining viable for over 4 weeks in culture [4] and murine PCLS remaining viable for over 60 days in culture [2]. However, despite maintaining viability for prolonged periods in culture, the integrity of the endothelial cells was lost by 7 days post slice in the murine PCLS; thus viability is not the only parameter to asses for the application of PCLS. Porcine PCLS have been repeatedly used in infection studies and were commonly analysed within 96 h [5, 11, 36, 37]. Ciliary activity and influenza virus titres [6] have been assessed in porcine PCLS for up to 7 days, but little data exists about the long term culture of porcine PCLS. As viability reduces around day 7 post slice in the avian PCLS model, it seems reasonable to recommend the use of the avian model within 7 days post slice.

Lung tissue is exposed to a wide range of microbial products and inert particles during inhalation, but despite this exposure, it generally maintains an immunologically quiescent state [38]. Accordingly, the threshold for the activation of antimicrobial responses is relatively high in lung tissue. Despite the quiescent nature of the tissue, PCLS generated from immunologically mature chickens, stimulated with LPS displayed a marked inflammatory response with secretion of type I IFN and IL-1β, as well as appreciable levels of nitric oxide breakdown products detected. Yet, these responses were only notable increased in response to LPS after 4 days in culture.

To investigate the observed delay in the onset of the inflammatory response, the anti-inflammatory cytokine IL-10, which is associated with tissue regeneration following injury [39], was examined in the PCLS. However, as IL-10 production displayed similar kinetics as pro-inflammatory molecules, it is likely that IL-10 is not mediating anti-inflammatory effects at the early time points following lung tissue slicing and damage. Nevertheless, other yet to be identified wound healing and tissue remodelling factors may account for this phenomenon. Transcriptional analysis of tracheal organ cultures identified altered immune responses during early culture, likely induced by the excision of the tissue, masking subsequent virus induced responses [40]. Reduced IL-1β and IL-6 levels were detected in the infected tracheal organ cultures, relative to infected trachea, at 24 h post infection, which is in agreement with the reduction in inflammatory response noted in the chicken PCLS during early culture. Other studies used tracheal organ cultures 5 days after preparation to avoid these initial responses [41]. Altogether, it is reasonable to assume that direct or cytokine-mediated tissue damage responses might be the cause of the early diminished responses observed in chicken PCLS.

Another potential mechanism accounting for reduced cytokine responses at early time points post slicing is the hypothermic condition at which the PCLS are prepared. The carcasses were chilled to 4 °C for up to 2 h to allow the agarose to set prior to slicing; and moreover, during the slicing procedure, the slices were maintained in chilled HBSS. Hypothermia has been shown to specifically inhibit cytokine responses of lung derived rat macrophages [42], with more generalised inhibitory effects on inflammatory cytokines and reactive oxygen species also reported in other mammalian cell types and organs [43, 44]. Despite the widely reported anti-inflammatory effects of hypothermia, there is little data available on the duration of this suppressive effect on pro-inflammatory cytokines on resuming normothermic conditions. However, the effects of 2 h of hypothermia, in terms of reduction of cellular infiltration, phagocytic potential and ICAM-1 expression, were still evident at 3 days post induction, in a rodent stroke model [45]. In line with this, our data point to days 4 to 7 as the optimal time frame for using chicken PCLS for studies on pro-inflammatory effects.

The previously described embryonic chicken PCLS were applied to examine the infectivity of different strains of IBV and to examine the cellular tropism of the virus [18]. Differential replication kinetics of a range of influenza A virus strains have been examined in porcine PCLS [46]. Here we tested for the first time whether PCLS from mature chickens could be permissive to avian influenza virus infection. The chicken PCLS were clearly susceptible to infections with the two LPAI virus strains tested. Foci of viral proteins within the PCLS, rather than diffuse areas of staining, indicated that the PCLS were readily infected by the two viruses, H7N1 and H1N1. Strikingly, this staining pattern resembles the virus-positive staining of lung tissues following LPAI virus infection in vivo [47].

Productive replication of the H7N1 virus was confirmed by an increased virus titre in the infected PCLS supernatants. The H7N1 titre at 4 h post-infection is likely to represent a remnant of the virus input, i.e. infectious viral particles that remained in the culture following the viral adsorption and washing steps. However, the 100-fold increase in virus titre at 24 h post-infection is indicative of productive replication of the virus in chicken PCLS, even though the titre dropped off at 48 h post-infection. NS1-positive staining was confirmed for the H1N1 virus up to 48 post-infection, pointing to efficient viral entry and the onset of viral replication. However, we did not detect any evidence for infectious H1N1 virus particle release in the supernatant above the level of the residual virus input. This lack of productive replication of the H1N1 virus was previously observed in chicken aortic endothelial cells and has been linked to the virus’ inability to escape the robust innate immune response in this cell type [32]. Therefore, it is an intriguing thought that similar mechanisms might also be responsible for the restricted replication of this virus in the PCLS from immunological mature chickens. Importantly, the differential replication properties of the H7N1 and H1N1 LPAI viruses in PCLS are in good agreement with previous data on viral (genome) loads in the lungs from live chickens infected with the two viruses [48, 49].

PCLS application in dynamic imaging studies has previously been demonstrated with the examination of dendritic cell antigen uptake and presentation to T cells in a murine model of allergic airway responses [50]; innate cell interaction between macrophages and dendritic cells during anthrax infection in mice [12]; and DC-T cell interactions in murine PCLS following in vivo injection of antigen specific T cells and instillation of cognate antigen [8]. Cellular interactions within the 3D structure of avian PCLS and the cells’ ability to sample and respond to the environment was confirmed in our studies. We demonstrated that immune cells, including phagocytes, are present in chicken PCLS, and that the interactions of pathogens with these cells can readily be examined by real-time dynamic imaging. Thus, the development of avian PCLS from immunologically mature donors offers a new opportunity to elucidate the initial interactions in the avian lung in the presence of heterogeneous cell populations and 3D architecture, evident in the intact organ. The importance of elucidating these early interactions in the avian lung is underscored by the preeminent role of the respiratory system in vaccine delivery in the poultry industry and its central role as entry port for respiratory pathogens. The use of PCLS as a model for examining early physiological and pathophysiological events in the lung therefore provides a novel tool in avian immunology to identify potential new and more efficient vaccine candidates or adjuvants as well as targets for antimicrobial therapeutics. The potential impact of applying this model in fundamental and applied research is highlighted by the global zoonotic threat posed by avian-origin influenza A viruses. The host response to influenza A viruses has been investigated in murine [51], porcine [46] and human PCLS [7]; however due to the central role of avian species in pandemic outbreaks and as a reservoir hosts, avian PCLS provide an invaluable research tool, which we have shown here to be permissive and/or susceptible to avian influenza virus infection. In the present study, we were able to stain endothelial cells within the PCLS by targeting a well conserved, multi-species target (vWF). Endotheliotropism is an exclusive trait of highly pathogenic avian influenza (HPAI) viruses in galliform birds and likely to be a critical determinant of viral pathogenicity [52, 53]. Hence, the chicken PCLS model presented here shall prove useful for studying important aspects of avian influenza virus pathogenicity and HPAI pathotype evolution in poultry.

In validating our PCLS model, we have unlocked the potential of utilising ex vivo tissue explants for a wide range of applications including visualizing immune cell dynamics, host–pathogen interactions and pathogen tropism, phenotyping of innate immune responses, testing of vaccine/adjuvant preparations and screening for antimicrobial activity. In addition, the role of endothelial cells in health and disease in the avian lung can also be investigated, with the first identification of this cell type in an organotypic preparation by vWF staining. We trust that the scientific community will greatly benefit from this thoroughly characterized PCLS model which permits addressing pertinent research questions in a complex avian organ system in full compliance with the 3Rs principles.

## Supplementary information

**Additional file 1. Live/Dead staining of PCLS.** PCLS (200–300 μm) prepared from PA12 SPF chickens and cultured in DMEM, DMEM/F12 or DMEM/F12/FCS. Representative images showing live (green) and dead (red) cells at day 1 (**A**), day 3 (**B**) and day 7 (**C**) post slice. Images were captured using a Zeiss Axiovert 200 M inverted epifluorescence microscope at ×200 magnification. Scale bars = 200 μm. Dead cells were enumerated in images from 3 to 4 independent PCLS (generated from a minimum of 2 individual birds) per time point and condition.

**Additional file 2. Limited effect of ambient temperature on PCLS viability.** PCLS (500 μm) were prepared from *CSF1R*-eGFP transgenic chickens and viability assessed by AlamarBlue assay over 40 days of culture at either 41 °C or 37 °C. Every 24 h the PCLS were incubated for 1 h with AlamarBlue reagent, the supernatants harvested and the fluorescence assessed in the supernatant relative to media incubated with AlamarBlue in the absence of PCLS. *n* = 6–14 slices prepared from 2 individual birds. Data are represented as the mean ± SD.

**Additional file 3. Mononuclear phagocytic cell motility in PCLS at 21** **days post slice.** eGFP-expressing mononuclear phagocytic cells (green) in PCLS (500 μm) generated from *CSF1R*-eGFP transgenic chickens were examined by time-lapse imaging using a Zeiss live cell observer with a 20× objective lens. The PCLS was imaging every 30 s for 1 h. The image was cropped to a region of interest demonstrating the motility of *CSF1R*-eGFP^+^ cells (green).

**Additional file 4. Additional controls for β-tubulin, NP and NS1 immunofluorescent staining of PCLS.** Isotype controls for β-tubulin as shown in Figure 3A. PCLS (200–300 μm) prepared from PA12 SPF chickens were cultured in DMEM/F12/FCS (**A**, panel i), DMEM/F12 (**A**, panel ii) or DMEM (**A**, panel iii), fixed and incubated with purified mouse IgG2a (Thermo Fischer Scientific, USA), the corresponding isotype control for the anti-β-tubulin antibody, followed by staining with goat anti-mouse IgG (H + L) Alexa Fluor 488 secondary antibody. Images were captured using a Leica TCS P8 confocal microscope at ×200 magnification. Scale bars = 75 µm. Images are representative of 3 independent PCLS per condition. Corresponding controls for PCLS (500 μm or 200–300 μm) infected with LPAI virus strains H7N1 or H1N1, respectively, show in Figure 7B. Immunofluorescent staining is shown for isotype control mouse IgG2a with secondary goat anti-mouse Ig FITC (B, panel i) and goat anti-mouse IgG-FITC secondary antibody alone (B, panel ii), which are the corresponding controls for the viral nucleoprotein (NP) staining of H7N1-infected PCLS. Immunofluorescent staining is shown for PCLS incubated with non-immunized rabbit serum (B, panel iii), the corresponding control for NS1 staining of H1N1-infected PCLS, followed by staining with goat anti-rabbit IgG (H + L) Alexa Fluor 488 secondary antibody. Images were captured using confocal microscopy at ×400 magnification. Scale bars = 10 µm (B, panels i and ii) and 25 µm (B, panel iii). All Images are representative of a minimum of 3 independent PCLS per condition, generated from a minimum of 2 individual birds.

**Additional file 5. Additional controls for Actin, von Willebrand Factor, MCRL1-B and CD45 immunofluorescent staining of PCLS.** Staining controls for Actin (**A**), von Willebrand Factor (**B**), MCRL1-B (**C)** and CD45 (**D**) immunofluorescent staining of PCLS (200–300 μm) as shown in Figure 4. PCLS prepared from SPF PA12 chickens 1 day post slice were fixed and incubated with: unlabelled phalloidin (**A**) (Abcam, UK), the corresponding control for the Rhodamine Phalloidin cytoskeleton staining; Purified rabbit IgG (Sigma-Aldrich, UK), the corresponding control for the von Willebrand Factor endothelial cells staining (**B**), followed by staining with goat anti-rabbit IgG (H + L) Alexa Fluor Plus 594 secondary antibody (Thermo Fisher Scientific, USA); Purified mouse IgG1 (Bio-Rad, UK), the corresponding control for the MCRL1-B^+^ monocytes/macrophages staining (**C**), followed by staining with goat anti-mouse IgG (H + L) Alexa Fluor 488 secondary antibody (Thermo Fisher Scientific, USA); and Purified mouse IgG2a (Bio-Rad, UK), the corresponding control for the CD45^+^ leukocytes staining (**D**), followed by staining with goat anti-mouse IgG (H + L) Alexa Fluor 488 secondary antibody (Thermo Fisher Scientific, USA). Images were captured using a Leica TCS P8 confocal microscope at ×200 magnification. Scale bars = 25 µm. All Images are representative of a minimum of 3 independent PCLS per condition, generated from a minimum of 2 individual birds.

**Additional file 6. Mononuclear phagocytic cell motility within the PCLS at 3** **days post slice.** PCLS (500 μm) prepared from *CSF1R*-eGFP transgenic chickens were incubated with fluorescent 1 μm latex beads (red) at 3 days post slice. Cellular motility and interactions were captured by time-lapse imaging using a Zeiss Live cell observer with a ×10 objective lens. The PCLS was imaged every 3 min for 18 h. The image was cropped to a region of interest demonstrating the motility and dynamic interactions of *CSF1R*-eGFP^+^ cells (green).

**Additional file 7. Dynamic interaction of mononuclear phagocytes with fluorescent latex beads.** PCLS (500 μm) prepared from *CSF1R*-eGFP transgenic chickens were incubated with 1 μm fluorescent latex beads at 3 days post slice, and were examined by time-lapse imaging using a Zeiss Live cell observer with a ×10 objective lens. The PCLS was imaged every 3 min for 18 h. The image was cropped to a region of interest demonstrating that *CSF1R*-eGFP^+^ cells (green) were phagocytosing latex beads (red).

**Additional file 8. Dynamic interactions of mononuclear phagocytes with APEC-eGFP.** PCLS (500 μm) prepared from *CSF1R*-mApple transgenic chickens were imaged at 3 days post slice using a Zeiss Live cell observer with a ×10 objective lens. The PCLS was imaged every 1 min for 1 h. The image was cropped to a region of interest demonstrating that *CSF1R*-mApple^+^ cells (red) were interacting with APEC-eGFP (green).

## Abbreviations

3D: 3-dimensional
APEC: avian pathogenic *Escherichia coli*
PCLS: precision-cut lung slice
LPS: lipopolysaccharide
LPAI: low pathogenic avian influenza
HPAI: highly pathogenic avian influenza
MNP: mononuclear phagocytes
IFN: interferon
FCS: fetal calf serum
CSF1R: colony stimulating factor 1 receptor
SPF: specific pathogen free
DMEM: Dulbecco’s modified Eagle medium
NP: nucleoprotein
NS1: non-structural protein 1
SD: standard deviation
