# Supplementary figures and images for: Precision cut lung slices: a novel versatile tool to examine host–pathogen interaction in the chicken lung
Source: Vet Res. 2020 Jan 10;51:2. doi: 10.1186/s13567-019-0733-0 (PMC6954617; doi:10.1186/s13567-019-0733-0)

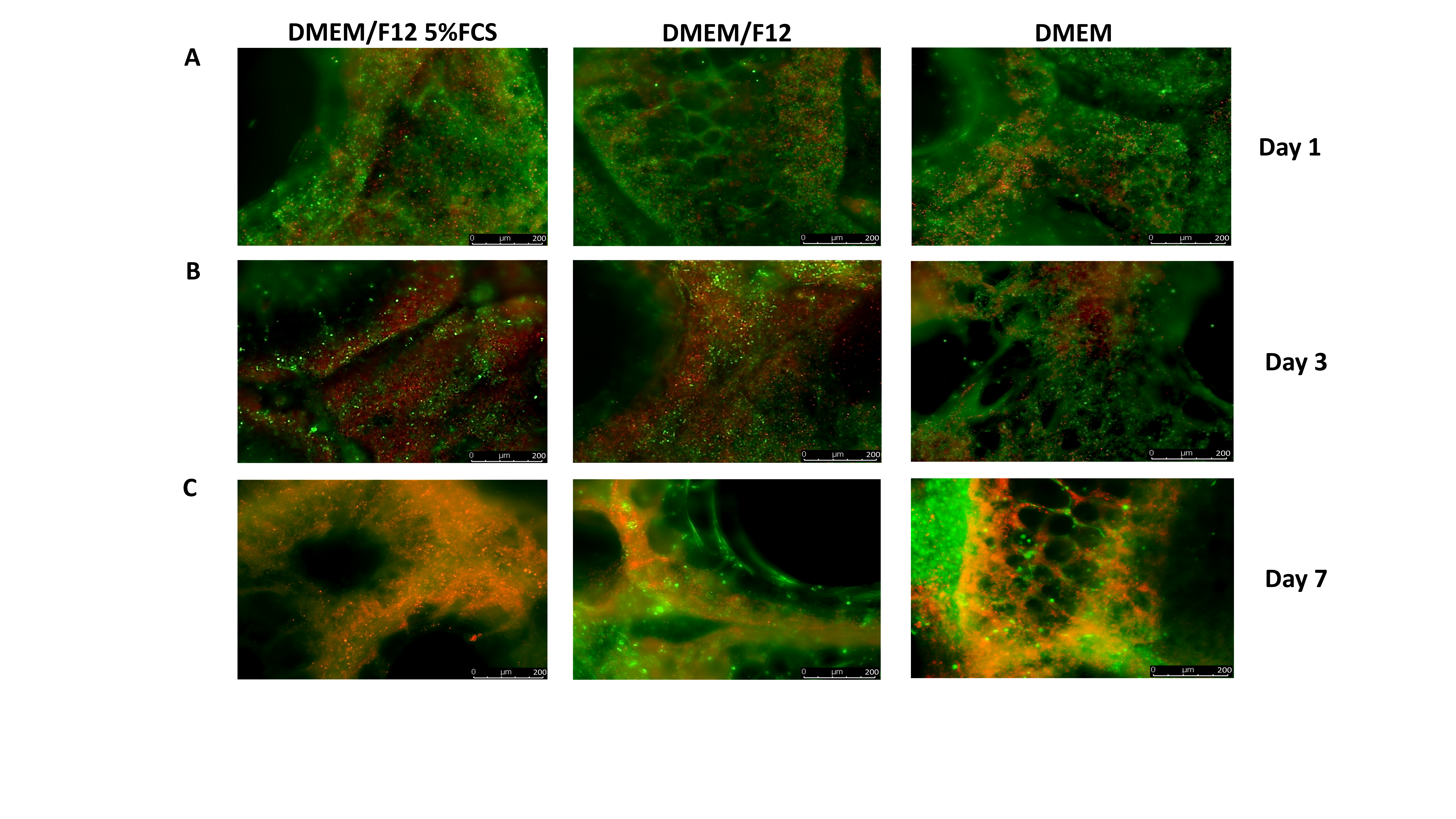

Supplement: Supplementary file 1 — Additional file 1. Live/Dead staining of PCLS. PCLS (200–300 μm) prepared from PA12 SPF chickens and cultured in DMEM, DMEM/F12 or DMEM/F12/FCS. Representative images showing live (green) and dead (red) cells at day 1 (A), day 3 (B) and day 7 (C) post slice. Images were captured using a Zeiss Axiovert 200 M inverted epifluorescence microscope at ×200 magnification. Scale bars = 200 μm. Dead cells were enumerated in images from 3 to 4 independent PCLS (generated from a minimum of 2 individual birds) per time point and condition. [file 13567_2019_733_MOESM1_ESM.tif]

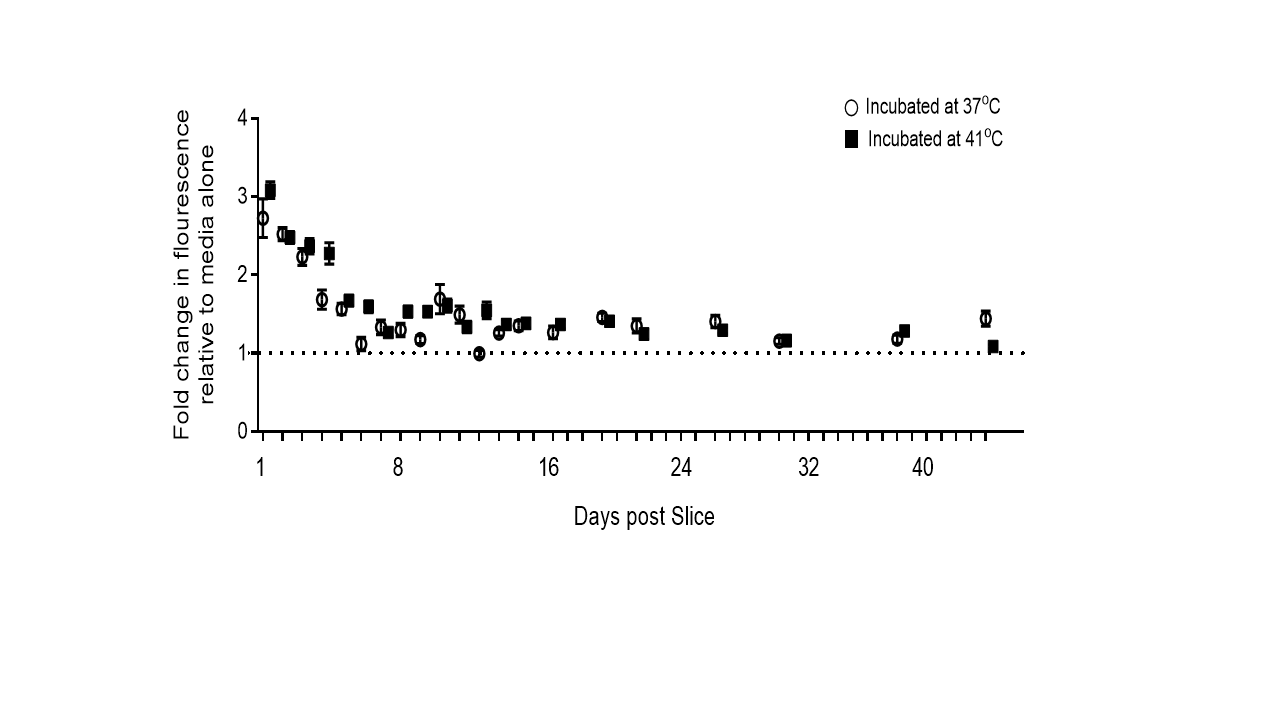

Supplement: Supplementary file 2 — Additional file 2. Limited effect of ambient temperature on PCLS viability. PCLS (500 μm) were prepared from CSF1R-eGFP transgenic chickens and viability assessed by AlamarBlue assay over 40 days of culture at either 41 °C or 37 °C. Every 24 h the PCLS were incubated for 1 h with AlamarBlue reagent, the supernatants harvested and the fluorescence assessed in the supernatant relative to media incubated with AlamarBlue in the absence of PCLS. n = 6–14 slices prepared from 2 individual birds. Data are represented as the mean ± SD. [file 13567_2019_733_MOESM2_ESM.tif]

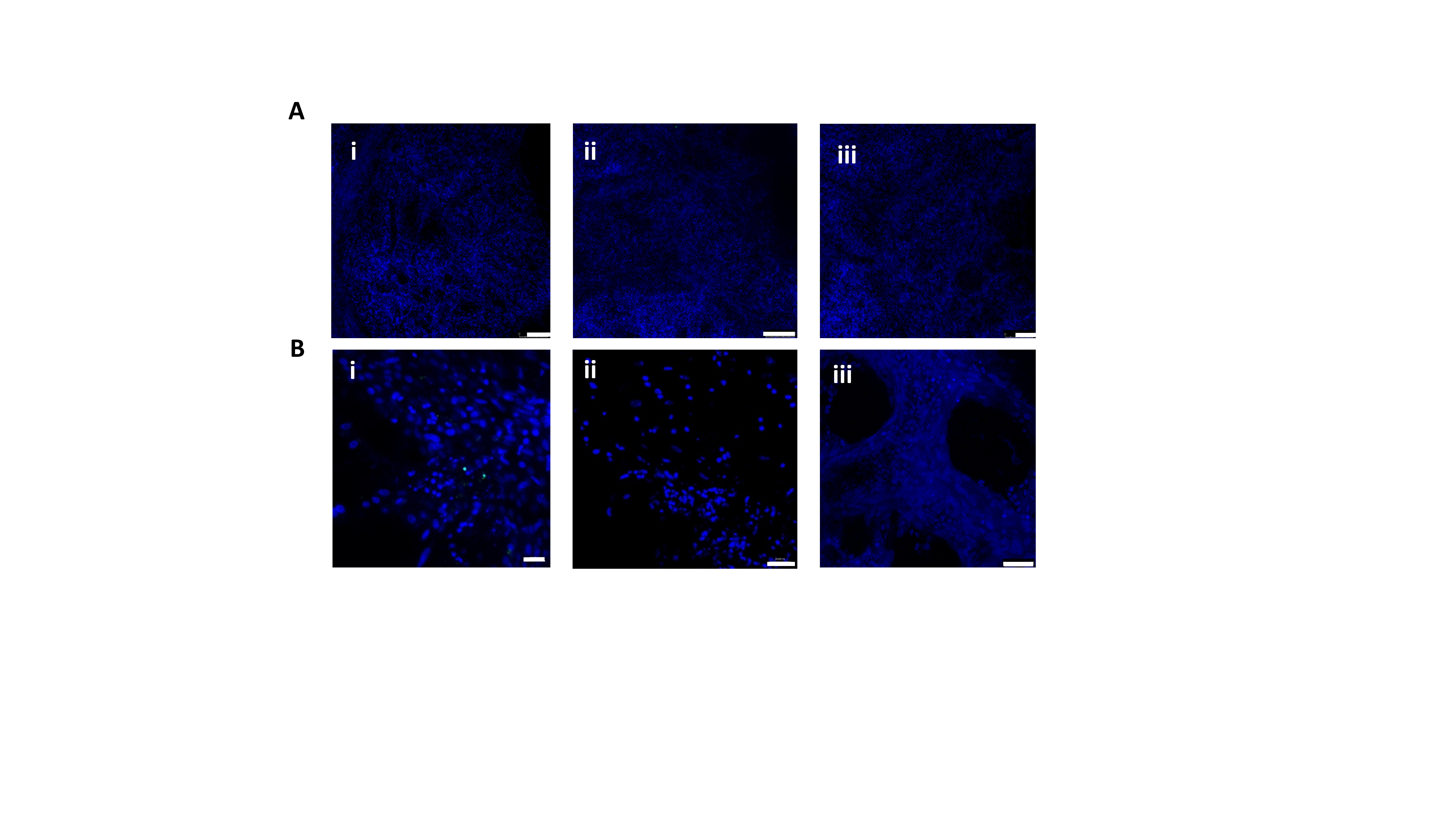

Supplement: Supplementary file 4 — Additional file 4. Additional controls for β-tubulin, NP and NS1 immunofluorescent staining of PCLS. Isotype controls for β-tubulin as shown in Figure 3A. PCLS (200–300 μm) prepared from PA12 SPF chickens were cultured in DMEM/F12/FCS (A, panel i), DMEM/F12 (A, panel ii) or DMEM (A, panel iii), fixed and incubated with purified mouse IgG2a (Thermo Fischer Scientific, USA), the corresponding isotype control for the anti-β-tubulin antibody, followed by staining with goat anti-mouse IgG (H + L) Alexa Fluor 488 secondary antibody. Images were captured using a Leica TCS P8 confocal microscope at ×200 magnification. Scale bars = 75 µm. Images are representative of 3 independent PCLS per condition. Corresponding controls for PCLS (500 μm or 200–300 μm) infected with LPAI virus strains H7N1 or H1N1, respectively, show in Figure 7B. Immunofluorescent staining is shown for isotype control mouse IgG2a with secondary goat anti-mouse Ig FITC (B, panel i) and goat anti-mouse IgG-FITC secondary antibody alone (B, panel ii), which are the corresponding controls for the viral nucleoprotein (NP) staining of H7N1-infected PCLS. Immunofluorescent staining is shown for PCLS incubated with non-immunized rabbit serum (B, panel iii), the corresponding control for NS1 staining of H1N1-infected PCLS, followed by staining with goat anti-rabbit IgG (H + L) Alexa Fluor 488 secondary antibody. Images were captured using confocal microscopy at ×400 magnification. Scale bars = 10 µm (B, panels i and ii) and 25 µm (B, panel iii). All Images are representative of a minimum of 3 independent PCLS per condition, generated from a minimum of 2 individual birds. [file 13567_2019_733_MOESM4_ESM.tif]

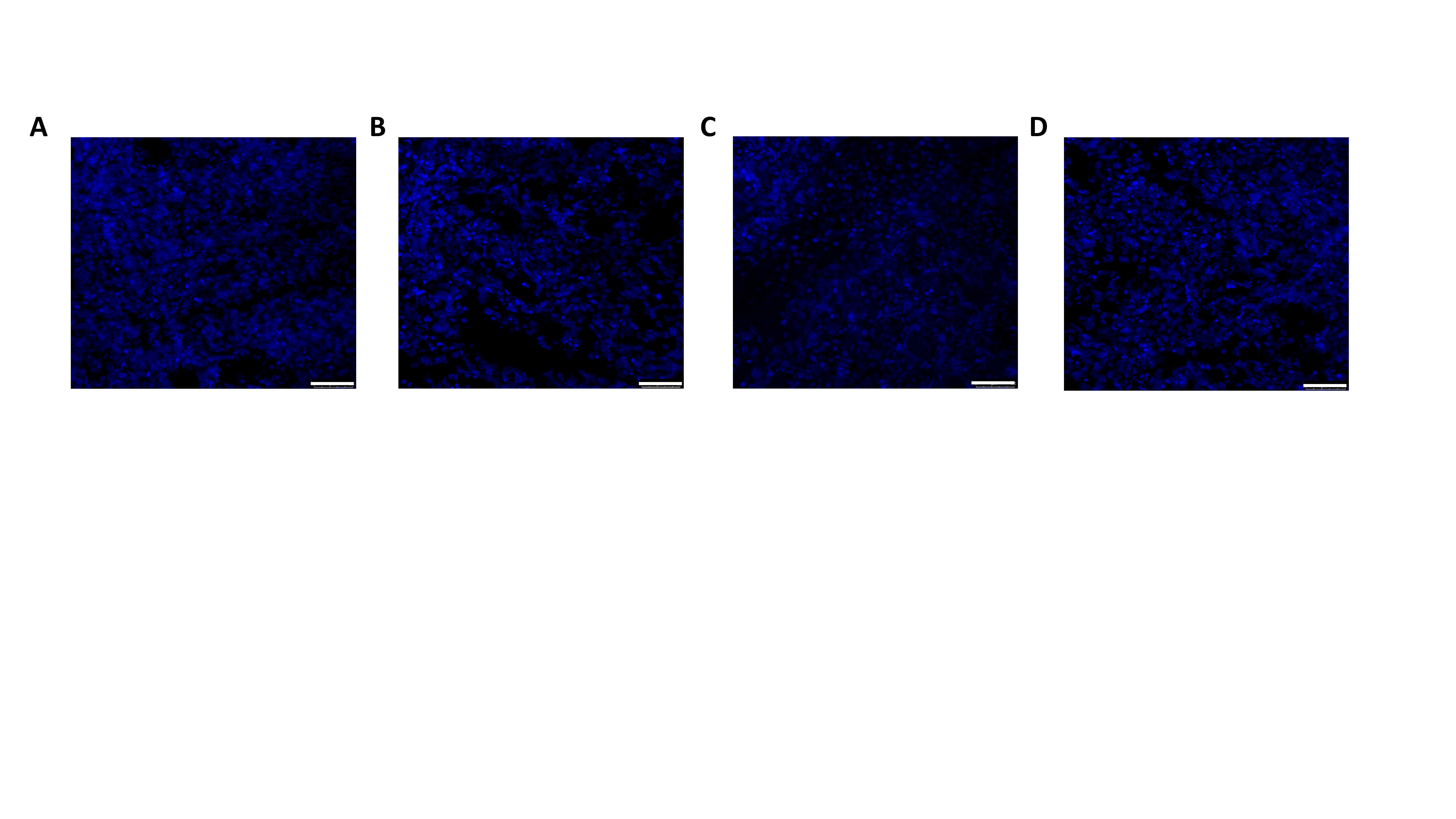

Supplement: Supplementary file 5 — Additional file 5. Additional controls for Actin, von Willebrand Factor, MCRL1-B and CD45 immunofluorescent staining of PCLS. Staining controls for Actin (A), von Willebrand Factor (B), MCRL1-B (C) and CD45 (D) immunofluorescent staining of PCLS (200–300 μm) as shown in Figure 4. PCLS prepared from SPF PA12 chickens 1 day post slice were fixed and incubated with: unlabelled phalloidin (A) (Abcam, UK), the corresponding control for the Rhodamine Phalloidin cytoskeleton staining; Purified rabbit IgG (Sigma-Aldrich, UK), the corresponding control for the von Willebrand Factor endothelial cells staining (B), followed by staining with goat anti-rabbit IgG (H + L) Alexa Fluor Plus 594 secondary antibody (Thermo Fisher Scientific, USA); Purified mouse IgG1 (Bio-Rad, UK), the corresponding control for the MCRL1-B+ monocytes/macrophages staining (C), followed by staining with goat anti-mouse IgG (H + L) Alexa Fluor 488 secondary antibody (Thermo Fisher Scientific, USA); and Purified mouse IgG2a (Bio-Rad, UK), the corresponding control for the CD45+ leukocytes staining (D), followed by staining with goat anti-mouse IgG (H + L) Alexa Fluor 488 secondary antibody (Thermo Fisher Scientific, USA). Images were captured using a Leica TCS P8 confocal microscope at ×200 magnification. Scale bars = 25 µm. All Images are representative of a minimum of 3 independent PCLS per condition, generated from a minimum of 2 individual birds. [file 13567_2019_733_MOESM5_ESM.tif]
